# Reusing pre-consumer textile waste

**DOI:** 10.1186/2193-1801-4-S2-O9

**Published:** 2015-11-27

**Authors:** Yuk-lan Lau

**Affiliations:** Department of Fashion and Image Design, Hong Kong Design Institute and Hong Kong Institute of Vocational Education (Lee Wai Lee), Hong Kong

## Background

With the expansion of the fashion industry the quantity of industrial pre-consumer textile waste has increased. It is estimated that approximately 10-20% of textiles are wasted during garment manufacture [1].

Reusing pre-consumer textile waste within the fashion supply chain offers many environmental advantages, including:

· Directing waste away from landfill and incinerators

· Conserving resources and providing a solution for the current shortages of natural resources and virgin fibres

· Providing low-cost raw materials for fashion products

· Delivering a lower monetary and environmental processing cost than virgin fibres

The reuse and recovery of textile waste causes only a fraction of the environmental, health and social damage caused by manufacturing the same amount of textiles from raw materials. Reclaiming fibres from textile waste avoids many of the polluting and energy intensive processes needed to make textiles from virgin materials.

As textiles are almost 100% recyclable, in an ideal world, nothing in the textile and apparel industry should be wasted. Recycling and reuse are therefore particularly important and must be addressed along the whole fashion supply chain. Pre-consumer textile waste is easier to reuse than post-consumer waste because it does not have the same hygiene and collection challenges.

In 2012 the Hong Kong Design Institute conducted research in collaboration with Redress on how to profitably reuse pre-consumer textile waste [2] in Hong Kong's fashion industry supply chain in ways that minimise negative environmental impacts. Their findings indicate that fashion and textile companies' concern for their bottom line determines their pre-consumer textile waste management practices.

## Methods

The primary research aimed to understand stakeholders' interests, practices and motivations regarding the reuse of textile waste. The five target groups were fashion designers, textile manufacturers, garment manufacturers, apparel retailers and consumers in Hong Kong and the Pearl River Delta economic region. Only companies with head offices in Hong Kong participated in the research; foreign companies with satellite offices in Hong Kong were excluded.

Surveys were conducted with 57 fashion designers, 48 textile manufacturers, 46 garment manufacturers, 64 retailers and 380 consumer respondents. Focus groups with between five and seven participants were held for each of the five target groups.

## Results

The survey results suggest that cost savings are the main motivators for reuse for stakeholders in Hong Kong's fashion supply chain. Surprisingly, the Hong Kong fashion and textile manufacturers are more driven to reduce textile waste than designers and retailers. A majority of consumers prefer sustainable clothing made using up-cycled pre-consumer textile waste, but they are not being offered enough access to desirable products. There appears to be a disconnection between consumer demand and the industry's perceived need for supply.

## Conclusions

To most effectively meet specific educational goals in international fashion sustainability studies, experiential learning approaches integrated the above research into the curriculum by initiating student projects, visiting the disadvantage groups in local communities, up-cycling pre-consumer textile waste into marketable products, launching products into the market and connecting students with the industry through internships in global fashion companies. Research into an experiential approach to sustainability studies [1] showed that experiential learning offers an educational experience that most effectively connects academic learning with practice, fosters an effective interdisciplinary curriculum, links students to work experience and job opportunities, and engages and empowers students.
